# Polygenic risk for neuropsychiatric disease and vulnerability to abnormal deep grey matter development

**DOI:** 10.1038/s41598-019-38957-1

**Published:** 2019-02-13

**Authors:** Harriet Cullen, Michelle L. Krishnan, Saskia Selzam, Gareth Ball, Alessia Visconti, Alka Saxena, Serena J. Counsell, Jo Hajnal, Gerome Breen, Robert Plomin, A. David Edwards

**Affiliations:** 1 Centre for the Developing Brain, School of Biomedical Engineering & Imaging Sciences, King’s College London, London, SE1 7EH United Kingdom; 2 Social, Genetic and Developmental Psychiatry Centre, Institute of Psychiatry, Psychology and Neuroscience, King’s College London, London, SE5 8AF United Kingdom; 3 Translational Medicine, Neuroscience and Rare Diseases, Roche Pharmaceutical Research and Early Development, Roche Innovation Centre, 4070 Basel, F. Hoffmann-La Roche, Ltd., Basel, Switzerland; 4 Developmental Imaging, Murdoch Children’s Research Institute, Melbourne, Australia; 5 Department of Twin Research and Genetic Epidemiology, King’s College London, London, SE1 7EH United Kingdom; 6 NIHR Biomedical Research Centre, Guy’s and St Thomas’ NHS Foundation Trust, London, SE1 9RT United Kingdom; 7 Department of Biomedical Engineering, School of Bioengineering and Imaging Sciences, King’s College London, London, SE1 7EH UK; 8 NIHR Biomedical Research Centre for Mental Health, South London and Maudsley NHS Trust, London, UK

## Abstract

Neuropsychiatric disease has polygenic determinants but is often precipitated by environmental pressures, including adverse perinatal events. However, the way in which genetic vulnerability and early-life adversity interact remains obscure. We hypothesised that the extreme environmental stress of prematurity would promote neuroanatomic abnormality in individuals genetically vulnerable to psychiatric disorders. In 194 unrelated infants (104 males, 90 females), born before 33 weeks of gestation (mean gestational age 29.7 weeks), we combined Magnetic Resonance Imaging with a polygenic risk score (PRS) for five psychiatric pathologies to test the prediction that: deep grey matter abnormalities frequently seen in preterm infants are associated with increased polygenic risk for psychiatric illness. The variance explained by the PRS in the relative volumes of four deep grey matter structures (caudate nucleus, thalamus, subthalamic nucleus and lentiform nucleus) was estimated using linear regression both for the full, mixed ancestral, cohort and a subsample of European infants. Psychiatric PRS was negatively associated with lentiform volume in the full cohort (β = −0.24, p = 8 × 10^−4^) and a European subsample (β = −0.24, p = 8 × 10^−3^). Genetic variants associated with neuropsychiatric disease increase vulnerability to abnormal lentiform development after perinatal stress and are associated with neuroanatomic changes in the perinatal period.

## Introduction

Preterm birth accounts for around 11% of all births and is a leading cause of infant mortality and morbidity^1^. It is a profound early-life stressor that is strongly associated with cognitive impairment, cerebral palsy, autism spectrum disorders and psychiatric disease^2–5^. Preterm birth is associated with both cerebral grey and white matter abnormalities. Research over the last decade has shown that the basal ganglia and thalamus are particularly vulnerable^6–10^. Boardman *et al*.^6^ showed the most marked morphological alteration between term and preterm infants to be a volume reduction in the thalamus and lentiform nucleus, a result confirmed by Srinivasan *et al*.^7^. Furthermore, this endophenotype has been associated with poorer neurodevelopmental outcome and neurodevelopmental disability^11,12^. We hypothesized that the neuroanatomical abnormality defined by these studies might be associated with vulnerability to the environmental stress of preterm birth and this vulnerability might be greater in individuals who are at increased genetic risk for psychiatric disorders.

Neuroanatomical and functional outcomes vary significantly between individuals and are likely to be modulated by the interaction of genetic and environmental factors^13,14^. Adverse psychiatric outcomes have moderate to high heritability^15^, as do many brain-imaging phenotypes^16–18^, even in early infancy^19^.

Recent research into the genetics of psychiatric disease has shown it to be polygenic: psychiatric disorders are influenced by many genetic variants each of which has an individually small effect^20^. As samples sizes have grown, genome wide association studies (GWAS) have become increasingly informative, allowing detection of small effects of single nucleotide polymorphisms (SNPs), although SNPs reaching the stringent statistical criteria for genome-wide significance (typically P < 5 × 10^−8^) are few and collectively explain only a small percentage of the genetic variance of the disorder in question^21^. However, it is now possible to generate individual-specific genotypic scores to predict phenotypic variance. GWAS results can be used to construct a polygenic risk score (PRS), which is an aggregate of trait-related effect sizes of SNPs across the genome in independent samples^22^.

We reasoned that if genes associated with psychiatric disease increase vulnerability to environmental effects on brain development, individuals with a greater polygenic risk for psychiatric disease who are subjected to the extreme environmental stress of preterm birth would be more likely to develop adverse consequences. Here, we exploit the power of previous large genome-wide studies measuring the polygenic risk for five different psychiatric disorders^23^, combining this with a large set of Magnetic Resonance Imaging (MRI) data in preterm infants to test the prediction that: the characteristic abnormalities seen in the basal ganglia and thalamus of preterm infants are associated with a greater polygenic risk for psychiatric illness.

## Results

Polygenic risk scores were computed at five different *P*-value thresholds (P_T_) for our sample of 194 preterm infants from summary statistics from the meta-analysis of genome-wide SNP data for five psychiatric disorders from the Cross-Disorder Group of the Psychiatric Genomics Consortium^23^. These were compared with structural MRI brain measures of four deep grey matter volumes for our cohort of preterm infants. Sample characteristics are given in Table 1.

**Table 1:** Summary statistics for three different ancestral cohorts.

Ancestry	Number of Subjects	Mean Gestational Age (weeks)	Mean Postmenstrual Age at Scan (weeks)	Mean Intracranial Volume (ml)
European	122	29.7 ± 0.2	42.3 ± 0.2	569.1 ± 5.8
Asian	48	29.6 ± 0.3	42.7 ± 0.3	557.1 ± 11.0
African	24	29.3 ± 0.6	43.4 ± 0.6	585.9 ± 16.0
Combined Sample	194	29.7 ± 0.2	42.6 ± 0.2	568.2 ± 4.9

**Figure 1:**
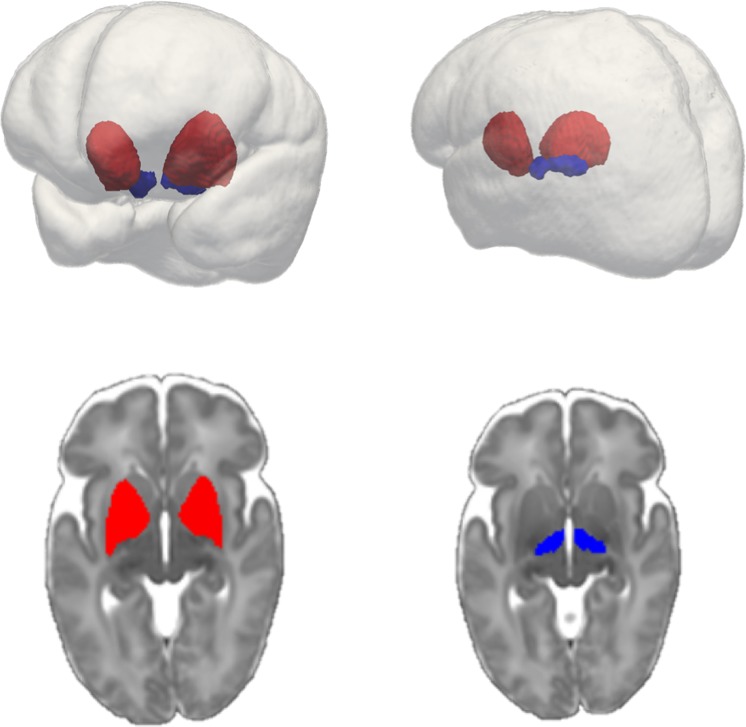
Subthalamic nucleus (blue) and Lentiform nucleus (red) within glass brain (top right and top left). Lentiform nucleus (bottom left) and subthalamic nucleus (bottom right) overlayed on 40-week neonatal template (axial cut).

**Figure 2:**
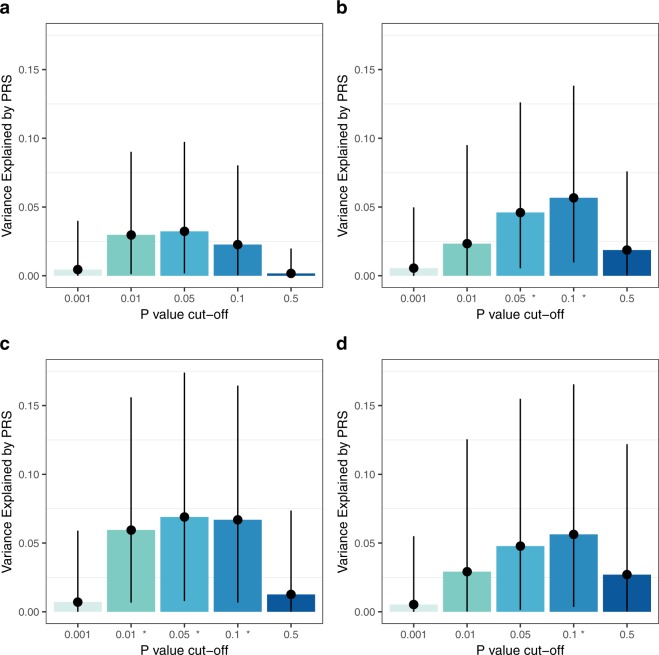
Proportion of variance explained in the subthalamic nucleus and lentiform nucleus volumes by the psychiatric PRS at five different *P*-value thresholds. Plots indicate the variance explained with estimated 95% confidence interval. The x-axis displays the five different upper thresholds of *P*-values for inclusion in the PRS. Results significant after multiple testing correction are indicated with an asterisk (*). (**a**) Subthalamic nucleus, full mixed-ancestral cohort, (**b**) Lentiform nucleus, full mixed-ancestral cohort, (**c**) Subthalamic nucleus, European sub-sample, (**d**) Lentiform nucleus, European sub-sample.

The psychiatric PRS predicted subthalamic and lentiform nucleus volumes in preterm infants in both the full mixed-ancestral cohort and the sub-sample of European infants. The subthalamic and lentiform nuclei are shown in Fig. 1. For the full, mixed-ancestry cohort, the psychiatric PRS was negatively associated with lentiform nuclear volume (β = −0.24, p = 8 × 10^−4^, R^2^ = 0.057, (p_T_ = 0.1)); the PRS also showed a modest negative association with subthalamic nuclear volume (β = −0.18, p = 0.01, R^2^ = 0.032, (p_T_ = 0.05)) which did not survive multiple testing correction (Table 2 and Fig. 2a and b). In the sub-sample of European infants, the psychiatric PRS was negatively associated with lentiform nuclear volume (β = −0.24, p = 8 × 10^−3^, R^2^ = 0.056, (p_T_ = 0.1)) and subthalamic nuclear volume (β = −0.26, p = 3 × 10^−3^, R^2^ = 0.069, (p_T_ = 0.05)) (Table 3 and Fig. 2c and d). For all associations with a p value < 0.05, the direction of the association was negative, that is, larger psychiatric genetic risk scores were associated with smaller deep grey matter volume. No association was found between the PRS and caudate or thalamic volume for either the full mixed-ancestral sample or the sub-sample of European infants (Tables 2 and 3). We note that these results remain robust if we correct the deep grey matter volumes for a more extended list of covariates that includes postmenstrual age at scan, gender and birth weight in addition to gestational age at birth and brain volume.

**Table 2:** Effect size and significance of correlations between psychiatric PRS and deep grey matter volumes for the full mixed-ancestral cohort. Standardised beta co-efficients and raw *P*-values are quoted. Raw *P*-values < 0.05 are shown in bold. Results surviving multiple-correction testing (p < 0.0083) are indicated with an asterisk (*).

Psychiatric genetic risk scores: SNPs with *P*-values <		Caudate Nucleus Volume	Thalamic Volume	Subthalamic Nucleus Volume	Lentiform Nucleus Volume
0.5	R^2^	0.007009	0.000452	0.001693	0.01871
	β	−0.08	0.02	−0.04	−0.14
	*P*-value	0.25	0.77	0.57	0.06
0.1	R^2^	0.01342	0.004909	**0.02266**	**0.0567**
	β	−0.12	−0.07	**−0.15**	**−0.24**
	*P*-value	0.11	0.33	**0.04**	0.0008*
0.05	R^2^	0.01417	0.004607	**0.03233**	**0.04602**
	β	−0.12	−0.07	**−0.18**	**−0.21**
	*P*-value	0.10	0.34	**0.01**	0.0027*
0.01	R^2^	0.005814	0.001811	**0.02966**	**0.02327**
	β	−0.08	−0.04	**−0.17**	**−0.15**
	*P*-value	0.29	0.56	**0.02**	**0.03**
0.001	R^2^	0.0106	0.000523	0.004517	0.005528
	β	−0.10	−0.02	−0.06	−0.07
	*P*-value	0.15	0.75	0.35	0.30

**Table 3:** Effect size and significance of correlations between psychiatric PRS and deep grey matter volumes for the European cohort. Standardised beta coefficients and raw *P*-values are quoted. Raw *P*-values < 0.05 are shown in bold. Results surviving multiple-correction testing (p < 0.0083) are indicated with *.

Psychiatric genetic risk scores: SNPs with *P*-values <		Caudate Nucleus Volume	Thalamic Volume	Subthalamic Nucleus Volume	Lentiform Nucleus Volume
0.5	R^2^	0.0114	8.451e-07	0.01263	0.02699
	β	−0.11	−0.001	−0.11	−0.16
	*P*-value	0.24	0.99	0.22	0.07
0.1	R^2^	0.01247	0.01219	**0.0669**	**0.05627**
	β	−0.11	−0.11	**−0.26**	**−0.24**
	*P*-value	0.22	0.23	0.004*	0.008*
0.05	R^2^	0.005469	0.003615	**0.06896**	**0.04785**
	β	−0.07	−0.06	**−0.26**	**−0.22**
	*P*-value	0.42	0.51	0.003*	**0.015**
0.01	R^2^	0.01572	0.004908	**0.05949**	0.02919
	β	−0.13	−0.07	**−0.24**	−0.17
	*P*-value	0.17	0.44	0.007*	0.06
0.001	R^2^	0.005688	8.784e-05	0.007118	0.005267
	β	−0.07	−0.01	−0.08	−0.07
	*P*-value	0.41	0.92	0.36	0.43

As an additional check, we looked for a possible relationship between our psychiatric PRS and gestational age at birth, no statistically significant relationship was found at any of the five *P*-value cut-offs.

## Discussion

We found evidence of an association between polygenic risk for psychiatric pathology and reduced lentiform volume in preterm infants. At its most predictive, in the full mixed-ancestry cohort, the psychiatric PRS explained approximately 6% of the variance in the lentiform nucleus volume. This is a comparatively large effect when compared with other work looking at psychiatric genetic risk and brain volume^24,25^.

We also found evidence for an association between our psychiatric PRS and reduced subthalamic volume in our European subsample however this result did not survive multiple-testing correction in our full mixed-ancestral cohort. Given this discrepancy, and the comparatively small volume of this region, it remains difficult to draw conclusions about the subthalamic nuclear result.

We found no statistically significant association between polygenic risk for psychiatric pathology and the volume of the thalamus or caudate nucleus. We were surprised not to find an association with the thalamus which has been consistently shown to be a vulnerable region following preterm birth^10^. Finally, results from an exploratory analysis using developmental outcome data suggest that the psychiatric PRS may be functionally significant as there was a modest negative association with expressive language, although this did not survive multiple testing correction.

Preterm infants have high levels of mental health problems and sub-diagnostic psychiatric symptomatologies, notably bipolar disorder, ADHD and ASD^5,26^; they also show characteristic abnormal deep grey matter development^6,7^ which is associated with adverse neurocognitive outcome^11,27^. This led us to hypothesise that common genetic risk variants which increase the risk of psychiatric pathology might also increase vulnerability to aberrant development of the basal ganglia and thalamus in individuals subjected to the unusual environmental stresses associated with preterm birth. This hypothesis may well be correct. However, we must also consider an alternative explanation: that genetic variants which increase the risk of psychiatric pathology are more ubiquitously associated with abnormal lentiform development and that this association is independent of the environmental precipitants associated with prematurity and would be similarly observed in a cohort of term-born infants or adults. In future work, we will seek to address this question using a cohort of both term and preterm infants.

We employed summary statistics from the work of Smoller *et al*.^23^ using results from their overall analysis combining five psychiatric disorders: autism spectrum disorder (ASD), attention deficit-hyperactivity disorder (ADHD), bipolar disorder, major depressive disorder, and schizophrenia. The empirical evidence of shared genetic aetiology for psychiatric disorders is strong. Lee *et al*.^28^ showed a strong genetic correlation between schizophrenia and bipolar disorder (0.68 ± 0.04 s.e.), and moderate between schizophrenia and major depressive disorder (0.43 ± 0.06 s.e.), bipolar disorder and major depressive disorder (0.47 ± 0.06 s.e.), and ADHD and major depressive disorder (0.32 ± 0.07 s.e.). The risk attributable to prematurity is high for all psychiatric disease, and also for sub-threshold generalised psychiatric symptomatology^5,26^. This commonalty in both symptomatology and genetic predisposition supports the use of a combined PRS in this study.

A number of studies have explored links between brain structure and genetic risk for psychiatric disorders in the adult population^25,29,30^. Terwisscha Van Scheltinga *et al*.^29^ found polygenic risk for schizophrenia was significantly associated with total brain volume. Other authors^25^ compared subcortical brain volume measures and PRS for schizophrenia and bipolar disorder in a sample of healthy adults; they found that most subcortical structures showed no association with a schizophrenia PRS with the exception of the globus pallidus and amygdala.

Xia *et al*.^24^ looked at genetic factors influencing global grey and white matter and intracranial volume in infants. They explored a possible association between genetic risk for both schizophrenia and ASD and global brain tissue volumes and found no association. They also integrated their GWAS results for global brain volumes with those for both adults and adolescents and found minimal overlap between common variants impacting brain volumes at different ages.

The most recent and largest adult study undertaken by Reus *et al*.^30^ looked at the impact of polygenic risk of major depressive disorder, schizophrenia, and bipolar disorder on subcortical brain volume and white matter microstructure. They found no statistically significant associations between either subcortical volumes or white matter microstructure and psychiatric polygenic risk, although they note a modest negative association between thalamic volume and the polygenic risk for schizophrenia. Franke *et al*.^31^ used a schizophrenia PRS and linkage disequilibrium (LD) score regression to test for shared genetic architecture between subcortical brain volume and schizophrenia and failed to find any overlap.

These large recent studies in adults have failed to show a robust association between deep grey matter volume and genetic psychiatric risk^30,31^. This makes our second explanation, that genetic variants associated with psychiatric risk are more generally associated with abnormal lentiform development, independent of environmental pressures, less likely. However, associations between brain volume and psychiatric risk might be more difficult to detect in older cohorts, or in cohorts where the environmental stressor is less extreme than preterm birth. Brain volumes in older individuals have been subject to variable environmental exposures for a far greater time than neonatal brains. Environmental effects, which include the influence of psychotropic medication^32^ may make genetic influences on brain structure more difficult to detect. It may be that the trajectory of growth of these structures is such that genetic overlap with psychiatric pathology is most easily detected early in life.

Studying preterm infants allows us to take a novel approach to the question of how genes that predispose to psychiatric disorders affect the brain at a neuroanatomical level. There is likely a strong interplay between environment and genetic determinants in psychiatric disease and preterm delivery is a major environmental stressor. Imaging-genomics studies of preterm infants might therefore uncover effects of risk genes on brain structure that have been hard to detect in adult and term-born infant studies.

This study used an imaging endophenotype extracted from a large imaging dataset which was homogeneous in terms of MRI acquisition protocol, data pre-processing, analysis and quality control. One challenge of this dataset was its ancestral diversity. The discovery GWAS sample used to derive the PRS was of European ancestry whereas our full cohort included infants of European, African and Asian origin. Excluding non-European infants would have significantly reduced our sample size and it is also important to generate growing amounts of evidence across a variety of common ancestral populations. We therefore undertook our analysis both in the full mixed-ancestral sample and performed an additional sensitivity analysis in the sub-sample of European babies. The general stability of results across the two cohorts is reassuring.

In summary, our study reports an association between volume of the lentiform nucleus in preterm infants and genetic risk for psychiatric pathology. Further annotation of the shared genetic architecture and its associated biological pathways may shed light on potential therapeutic strategies.

## Methods and Materials

### Subjects

Preterm infants were recruited as part of the EPRIME (Evaluation of Magnetic Resonance (MR) Imaging to Predict Neurodevelopmental Impairment in Preterm Infants) study and were imaged at term equivalent age. The EPRIME study was conducted according to the principles of the Declaration of Helsinki, and ethical approval was obtained from the UK National Research Ethics Service. Written parental informed consent was obtained for all subjects.

### Imaging

#### MRI acquisition

MRI was performed on a Philips 3-Tesla system (Philips Medical Systems, Best, The Netherlands) within the Neonatal Intensive Care Unit using an 8-channel phased array head coil.

T1-weighted MRI was acquired using: repetition time (TR): 17 ms; echo time (TE): 4.6 ms; flip angle 13°; slice thickness: 0.8 mm; field of view: 210 mm; matrix: 256 × 256 (voxel size: 0.82 × 0.82 × 0.8 mm). T2-weighted fast-spin echo MRI was acquired using: TR: 14730 ms; TE: 160 ms; flip angle 90°; field-of-view: 220 mm; matrix: 256 × 256 (voxel size: 0.86 × 0.86 × 2 mm) with 1 mm overlap.

Pulse oximetry, temperature and heart rate were monitored throughout and ear protection was used for each infant (President Putty, Coltene Whaledent, Mahwah, NJ; MiniMuffs, Natus Medical Inc, San Carlos, CA).

#### Image processing

T1-weighted images were brain-extracted with co-registered T2 brain masks (FSL’s BET; FSL 5.0.8, http://fsl.fmrib.ox.ac.uk/fsl) and corrected for bias field inhomogeneities^33^. Each subjects’ T1-weighted image was aligned to a 40-week neonatal template^34^ using nonlinear registration^35^. Voxelwise maps of volume change induced by the transformation were characterized by the determinant of the Jacobian operator, referred to here as the Jacobian map. These maps include a global scaling factor, so Jacobian values reflect tissue volume differences due to both local deformation and global head size. T1-derived Jacobian maps were iteratively smoothed to a FWHM of 8 mm (AFNI’s 3dBlurToFWHM; http://afni.nimh.nih.gov/afni). Each map was then log-transformed so that values greater than zero represent local areal expansion in the subject relative to the target and values less than zero represent areal contraction.

#### Brain Endophenotype

The Jacobian maps were used to estimate the volume of regions of interest in the deep grey matter. Volume estimates for the bilateral volumes of the thalamus, subthalamic nucleus, caudate nucleus and lentiform nucleus (putamen and globus pallidus) were extracted by computing the mean log(Jacobian) within each region-specific mask. Masks were defined using a neonatal atlas^36^ aligned to a 40-week template.

#### Genotyping and quality control

348 saliva samples were collected using Oragene DNA OG-250 kits (DNAGenotek Inc., Kanata, Canada) and genotyped on Illumina HumanOmniExpress-24 v1.1 arrays (Illumina, San Diego, CA, USA). Individuals with genotyping completeness <95% were excluded (29 individuals removed). SNPs with a minor allele frequency <0.01 (24546 SNPs), a missing genotype rate <99% (14672 SNPs) and deviations from Hardy–Weinberg equilibrium, P < 1 × 10^−7^ (2307 SNPs) were removed. Where pairs with high levels of relatedness existed (pi-hat > 0.3) only one member of each pair was retained (44 individuals removed). This resulted in a sample of 275 individuals with high-quality genetic data (635266 SNPs). All quality control steps were carried out using PLINK 1.9^37^ (Software: https://www.cog-genomics.org/plink/1.9/).

### Population Stratification and Sample Selection

After pruning to remove markers in high linkage disequilibrium (r^2^ > 0.1, 72900 SNPs retained) we performed a Principal Component Analysis using PLINK 1.9^38^. Inspection of the first two principal components in combination with reported ethnicity was used to define three ancestral populations: European, Asian and African (Supplementary Figure S1). Outliers from these three ancestral populations were excluded (35 individual removed). These three populations formed the cohorts for ongoing analysis (240 individuals).

Only those individuals with high quality MRI T1 data were retained (214 of the 240). Infants with major focal lesions such as periventricular leukomalacia, hemorrhagic parenchymal infarction and other ischaemic or haemorrhagic lesions were excluded from the analysis (20 infants). Supplementary Table S2 (Supplementary Information) gives further details of the focal brain lesions of the infants excluded. Our final sample comprised of 194 unrelated preterm infants (104 males, 90 females), mean gestational age 29.7 weeks, mean postmenstrual age at scan 42.6 weeks, including 122 individuals in the European cohort, 48 in the Asian cohort and 24 in the African cohort (Table 1).

### Polygenic Scoring

We computed polygenic risk scores (PRS) for the 194 unrelated individuals using odds ratios and *P*-values from summary statistics obtained by the primary GWAS analysis from the Cross-Disorder Group of the Psychiatric Genomics Consortium^23^. This analysis combined the effects of five psychiatric diseases (autism spectrum disorder, attention deficit-hyperactivity disorder, bipolar disorder, major depressive disorder, and schizophrenia) in 33332 cases and 27888 controls of European ancestry. The authors used a meta-analytic approach that applied a weighted Z-score with weights indicating the sample-size of the disease specific studies. The greatest power was therefore for SNPs that have an effect in multiple disorders.

PRS were generated in PRSice^39^. Initially the three ancestral sub-samples were considered independently. Quality-controlled SNPs were pruned for linkage disequilibrium based on *P*-value informed clumping using a r^2^ = 0.1 cut-off within a 250-kb window to create a SNP-set in linkage equilibrium for each of our ancestral cohorts. The MHC region was not removed in computation of the PRS, removal does not materially affect the results. PRS were computed at five different *P*-value thresholds (P_T_) in the discovery GWAS summary statistics: 0.001, 0.01, 0.05, 0.1, 0.5. Scores were computed using genotyped SNPs in our target dataset. *P*-value thresholds and the number of SNPs contributing to the PRS for each of the three ancestral subsamples at each threshold are summarized in Supplementary Table S1.

To control for population stratification, we regressed the PRS on the first 10 principal components of our ancestry matrix and used the residuals in all subsequent analysis. Details of the effect of this regression on the PRS distributions for our three ancestral cohorts are outlined in the supplementary information: Supplementary Methods and Supplementary Figures S2 and S3.

### Statistical Analysis

Bilateral deep grey matter brain volumes for the thalamus, subthalamic nucleus, caudate nucleus and lentiform nucleus (globus pallidus and putamen) were corrected for intracranial volume and gestational age at birth. Corrected volumes were then regressed on the ancestry-corrected PRS generated at five different *P*-value thresholds using simple linear regression in R. The variance explained by the PRS was obtained by squaring the regression coefficient.

Our full sample includes infants from three different ancestral cohorts: European, Asian and African. In contrast, the GWAS meta-analysis results^23^ used to compute the PRS were compiled using subjects of European ancestry only. We have therefore conducted an additional sensitivity analysis using a sub-sample of our cohort comprising only the European infants.

Preterm birth is known to be associated with an increased risk of psychiatric disease. We therefore checked for a possible association between the psychiatric PRS and degree of prematurity (gestational age at birth). We additionally undertook two exploratory analyses. We looked for a possible relationship between psychiatric PRS and developmental outcome and psychiatric PRS and intracranial volume (Supplementary Methods and Results).

Results quoted in Tables 2 and 3 are raw P-values. We have additionally computed a multiple-comparison *P*-value threshold. Since both the deep grey matter volumes and the five polygenic risk score thresholds are highly correlated we have used the method proposed by^40^ to compute the effective number of independent tests performed (M_eff_). This accounts for the correlation structure between measures and calculates the M_eff_ based on the observed eigenvalue variance using the matSpD interface (https://gump.qimr.edu.au/general/daleN/matSpD/). This calculation was performed for both the deep grey matter volumes (M_eff_dgm_) and the differently-thresholded polygenic scores (M_eff_prs_). The *P*-value for significance was determined as 0.05 divided by the product of M_eff_dgm_ and M_eff_prs_. The four deep grey matter volumes resulted in two independent tests and the five differently-thresholded polygenic scores three independent tests, giving a multiple-comparison corrected *P*-value threshold of p < 0.008333. Results that survive the multiple-comparison correction in Tables 2 and 3 are indicated with an asterisk (*).

## Supplementary information

Supplementary Information

## Data Availability

Data are available from the authors upon request, subject to approval of future uses by the National Research Ethics Service. The study was approved by the UK National Research Ethics Service, and written parental informed consent was obtained for all subjects.

## References

[1] Blencowe H (2012). National, regional, and worldwide estimates of preterm birth rates in the year 2010 with time trends since 1990 for selected countries: A systematic analysis and implications. Lancet.

[2] Moore T (2012). Neurological and developmental outcome in extremely preterm children born in England in 1995 and 2006: the EPICure studies. Bmj.

[3] Mackay DF, Smith GCS, Dobbie R, Pell JP (2010). Gestational age at delivery and special educational need: Retrospective cohort study of 407,503 schoolchildren. PLoS Med..

[4] Johnson S, Marlow N (2011). Preterm birth and childhood psychiatric disorders. Pediatr. Res..

[5] Nosarti C (2012). Preterm birth and psychiatric disorders in young adult life. Arch. Gen. Psychiatry.

[6] Boardman JP (2006). Abnormal deep grey matter development following preterm birth detected using deformation-based morphometry. Neuroimage.

[7] Srinivasan, L., Dutta, R., Counsell, S. J., Allsop, J. M. & Boardman, J. P. Quantification of Deep Gray Matter in Preterm Infants at Term-Equivalent Age Using Manual Volumetry of 3-Tesla Magnetic Resonance Images. **119** (2007). 10.1542/peds.2006-2508 17403847

[8] Ball G (2012). The effect of preterm birth on thalamic and cortical development. Cereb. Cortex.

[9] Ligam P (2009). Thalamic damage in periventricular leukomalacia: Novel pathologic observations relevant to cognitive deficits in survivors of prematurity. Pediatr. Res..

[10] Volpe JJ (2009). Brain injury in premature infants: a complex amalgam of destructive and developmental disturbances. Lancet Neurol..

[11] Inder, T. E., Warfield, S. K., Wang, H. & Hu, P. S. Abnormal Cerebral Structure Is Present at Term in Premature Infants. **115** (2005). 10.1542/peds.2004-0326 15687434

[12] Ball, G. *et al*. Multimodal image analysis of clinical influences on preterm brain development. *Ann. Neurol*. 233–246, 10.1002/ana.24995 (2017). 10.1002/ana.24995 PMC5601217 28719076

[13] Dempfle A (2008). Gene-environment interactions for complex traits: definitions, methodological requirements and challenges. Eur. J. Hum. Genet..

[14] Leviton A, Gressens P, Wolkenhauer O, Dammann O (2015). Systems approach to the study of brain damage in the very preterm newborn. Front Syst Neurosci.

[15] Polderman TJC (2015). Meta-analysis of the heritability of human traits based on fifty years of twin studies. Nat. Genet..

[16] Kochunov P (2015). Heritability of fractional anisotropy in human white matter: a comparison of Human Connectome Project and ENIGMA-DTI data. Neuroimage.

[17] Schmitt JE (2010). A twin study of intracerebral volumetric relationships. Behav. Genet..

[18] Den Braber A (2013). Heritability of subcortical brain measures: A perspective for future genome-wide association studies. Neuroimage.

[19] Gilmore JH (2010). Genetic and environmental contributions to neonatal brain structure: A twin study. Hum. Brain Mapp..

[20] Wray NR (2014). Research Review: Polygenic methods and their application to psychiatric traits. J. Child Psychol. Psychiatry Allied Discip..

[21] Manolio TA (2009). Finding the missing heritability of complex diseases. Nature.

[22] Dudbridge, F. Power and Predictive Accuracy of Polygenic Risk Scores. *PLoS Genet*. **9** (2013). 10.1371/journal.pgen.1003348 PMC3605113 23555274

[23] Smoller JW (2013). Identification of risk loci with shared eff ects on five major psychiatric disorders: A genome-wide analysis. Lancet.

[24] Xia K (2017). Genome-wide association analysis identifies common variants influencing infant brain volumes. Transl. Psychiatry.

[25] Caseras X, Tansey KE, Foley S, Linden D (2015). Association between genetic risk scoring for schizophrenia and bipolar disorder with regional subcortical volumes. Transl. Psychiatry.

[26] Kroll J (2018). A dimensional approach to assessing psychiatric risk in adults born very preterm. Psychol. Med..

[27] Boardman JP (2010). A common neonatal image phenotype predicts adverse neurodevelopmental outcome in children born preterm. Neuroimage.

[28] Lee SH (2013). Genetic relationship between five psychiatric disorders estimated from genome-wide SNPs. Nat. Genet..

[29] Terwisscha Van Scheltinga AF (2013). Genetic schizophrenia risk variants jointly modulate total brain and white matter volume. Biol. Psychiatry.

[30] Reus, L. M. *et al*. Association of polygenic risk for major psychiatric illness with subcortical volumes and white matter integrity in UK Biobank. *Nat. Publ. Gr*. 1–8, 10.1038/srep42140 (2017). 10.1038/srep42140 PMC5301496 28186152

[31] Franke B (2016). Genetic influences on schizophrenia and subcortical brain volumes: large-scale proof of concept. Nat. Neurosci..

[32] Moncrieff J, Leo J (2010). A systematic review of the effects of antipsychotic drugs on brain volume. Psychol. Med..

[33] Tustison NJ (2010). N4ITK: Improved N3 Bias Correction. IEEE Trans. Med. Imaging.

[34] Serag A (2012). Construction of a consistent high-definition spatio-temporal atlas of the developing brain using adaptive kernel regression. Neuroimage.

[35] Rueckert D, Frangi AF, Schnabel JA (2003). Automatic construction of 3-D statistical deformation models of the brain using nonrigid registration. Med. Imaging, IEEE Trans..

[36] Makropoulos A (2016). Regional growth and atlasing of the developing human brain. Neuroimage.

[37] Purcell S (2007). PLINK: A Tool Set for Whole-Genome Association and Population-Based Linkage Analyses. Am. J. Hum. Genet..

[38] Chang CC (2015). Second-generation PLINK: rising to the challenge of larger and richer datasets. Gigascience.

[39] Euesden J, Lewis CM, O’Reilly PF (2014). PRSice: Polygenic Risk Score software. Bioinformatics.

[40] Li J, Ji L (2005). Adjusting multiple testing in multilocus analyses using the eigenvalues of a correlation matrix. Heredity (Edinb)..

