# Supplementary material for: Polygenic risk for neuropsychiatric disease and vulnerability to abnormal deep grey matter development
Source: Sci Rep. 2019 Feb 13;9:1976. doi: 10.1038/s41598-019-38957-1 (PMC6374514; doi:10.1038/s41598-019-38957-1)
Supplement: Supplementary file 1 — Supplementary Information [file 41598_2019_38957_MOESM1_ESM.docx]

Supplementary Information for:

**Polygenic risk for neuropsychiatric disease and vulnerability to abnormal deep grey matter development**

Harriet Cullen^1^, Michelle L Krishnan^1,3^, Saskia Selzam^2^, Gareth Ball^1,4^, Alessia Visconti^5^, Alka Saxena^6^, Serena J Counsell^1^, Jo Hajnal^1^, Gerome Breen^2^, Robert Plomin^2^, A David Edwards^1^

^1^ Centre for the Developing Brain, Kings College, London, SE1 7EH, United Kingdom,

^2^ Institute of Psychiatry, Psychology and Neuroscience, Kings College London, SE5 8AF, United Kingdom,

^3^ Translational Medicine, Neuroscience and Rare Diseases, Roche Pharmaceutical Research and Early Development, Roche Innovation Center, 4070 Basel, F. Hoffmann-La Roche, Ltd.

^4^Developmental Imaging, Murdoch Children’s Research Institute, Melbourne, Australia

^5^Department of Twin Research and Genetic Epidemiology, King's College London, SE1 7EH United Kingdom

^6^NIHR Biomedical Research Center, Guy's and St Thomas' NHS Foundation Trust, London SE1 9RT, United Kingdom

**Correspondence author:** Harriet Cullen

Email: harriet.cullen@kcl.ac.uk

**This file includes:**

**Supplementary Text**: Supplementary Methods, Supplementary Results

**Supplementary Figures**: Figures. S1 to S3

**Supplementary Tables**: Table S1 and Table S2

**Supplementary Information Text**

**Supplementary Methods: Population Stratification**

To control for population stratification, we regressed the psychiatric polygenic risk score (PRS) on the first 10 principal components of our ancestry matrix and used the residuals in all subsequent analysis. The effect of this regression is illustrated in **Supplementary** **Figure S2.** and **Supplementary Figure S3**. Prior to correction for ancestry there is a statistically significant difference between the psychiatric PRS means of all three populations: European and African population (p = 5.998e-09), European and Asian populations (p = 2.2e-16) and the African and Asian populations (p = 2.2e-16) (**Supplementary** **Figure S2**). Following correction of the psychiatric PRS for ancestry there is no statistically significant difference in the means of the PRS score for any population: European and African populations (p = 1), European and Asian populations (p = 1), and the African and Asian populations (p =1) (**Supplementary** **Figure S3**).

**Supplementary Methods and Results: Developmental Outcome and Intracranial Brain Volume**

We undertook two explorational analyses. We looked for a possible relationship between our psychiatric PRS and developmental outcome. Of 194 subjects who had suitable MRI data all returned for neurodevelopmental assessment at a median age of 20.18 months corrected age (mean of 20.48 months corrected age). Neurodevelopmental performance was assessed using the Bayley Scales of Infants and Toddler Development, Third Edition (42) and cognitive, language and motor composite scores were obtained. A modest negative association was observed between Expressive Communication and psychiatric PRS (p = 0.013, β =-0.18, (P_T_=0.01)).

We also looked for a possible relationship between our psychiatric PRS and intracranial volume. We found no statistically significant relationship between the psychiatric genetic risk score and intracranial brain volume (corrected for gestational age) in the full mixed-ancestral cohort or the European sub-sample (p values for all P_T_ thresholds > 0.05 in both samples).

**Supplementary Figures, Tables and Legends**

**Supplementary Figure S1.**


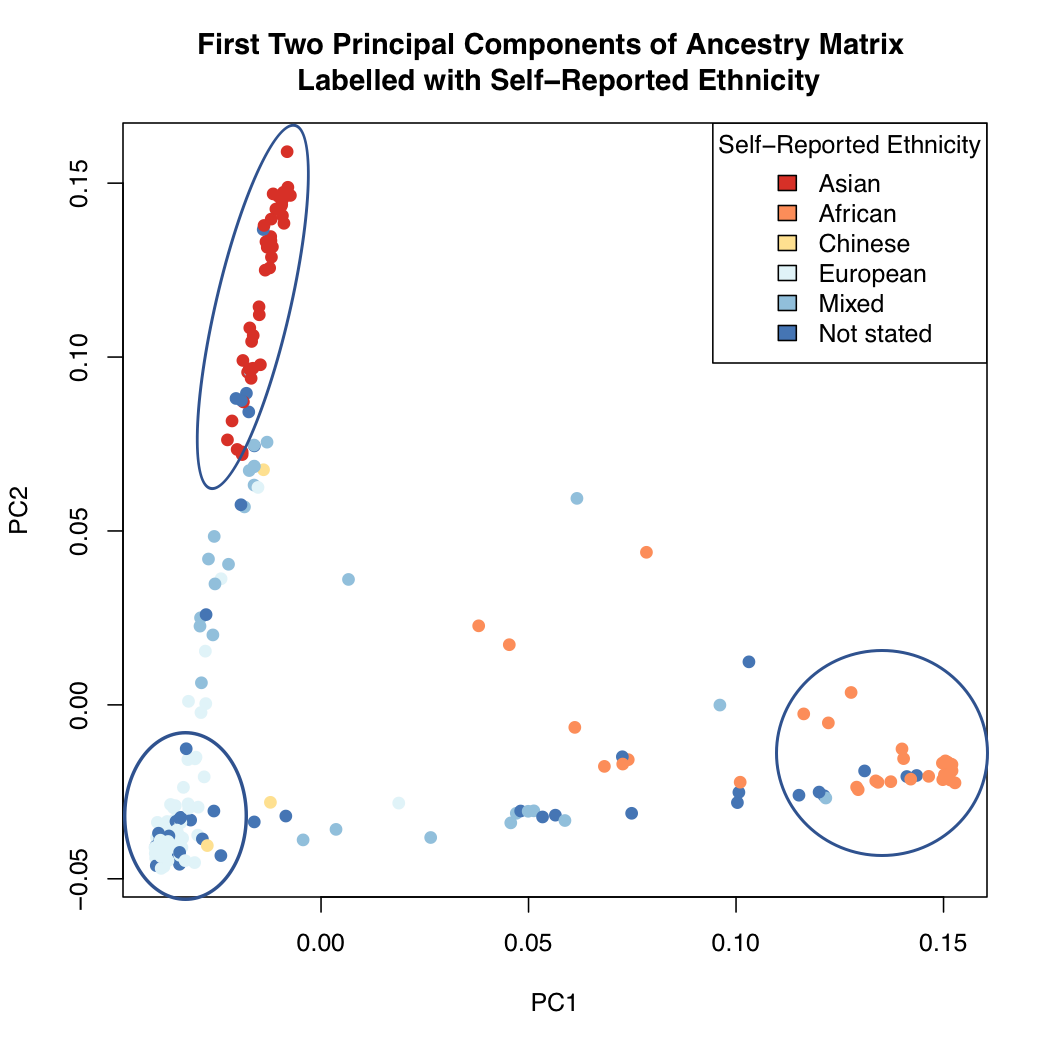


**Supplementary Figure S1.** A plot of the first two principle components of the ancestry matrix color labelled with self-reported ethnicity. Three ancestral populations, indicated with circles, were identified that were then used to explore possible association between deep grey matter brain volume and psychiatric polygenic risk (top left Asian, bottom left European, bottom right African).

**Supplementary Figure S2.**


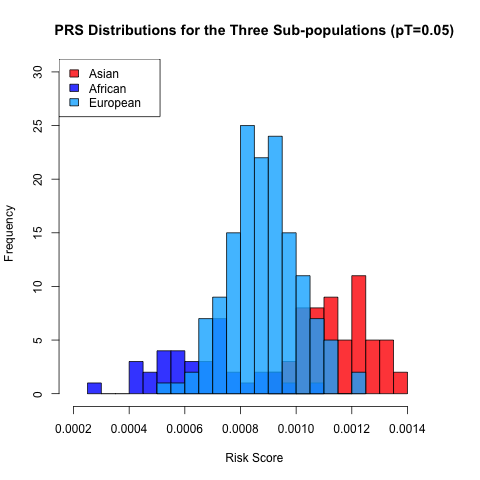


**Supplementary Figure S2.** Distribution of raw psychiatric PRS (*P*-value threshold P_T_ = 0.05) for the three sub-populations before correction for ancestry. There is a statistically significant difference between the means of the PRS for the three different populations.

**Supplementary Figure S3.**


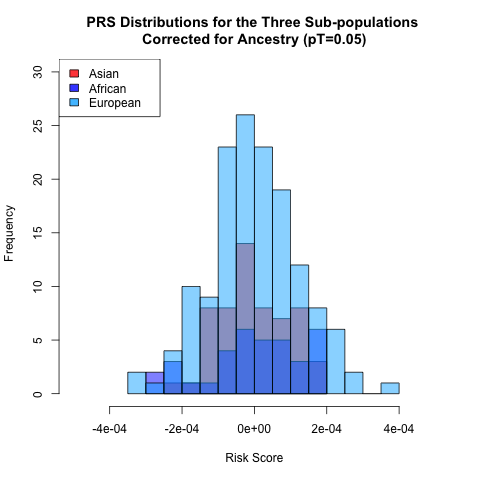


**Supplementary Figure S3.** Distribution of psychiatric PRS (*P*-value threshold P_T_ = 0.05) following correction for ancestry with the first ten principle components of the ancestry matrix. There is no statistically significant difference between the means of the PRS for any of the three populations.

Supplementary Table S1.

| **P-value threshold** | **European** | **Asian** | **African** | **Combined Mean** |
| --- | --- | --- | --- | --- |
| **0.001** | 698 | 756 | 999 | 818 |
| **0.01** | 3891 | 4127 | 5164 | 4394 |
| **0.05** | 13234 | 13827 | 17188 | 14750 |
| **0.1** | 22282 | 23122 | 28144 | 24516 |
| **0.5** | 69647 | 68149 | 77306 | 71700 |
| **All SNPs** | 102602 | 98830 | 110083 | 103838 |

**Supplementary Table S1** The number of SNPs included in the polygenic risk scores for each of the three ancestral subsamples at the five different P-value thresholds.

**Supplementary Table S2.**

| Lesions | Number (%) |
| --- | --- |
| Periventricular leukomalacia | 8 (40%) |
| Hemorrhagic parenchymal infarction | 6 (30%) |
| Multiple cerebellar hemorrhages or atrophy | 1 (5%) |
| Thalamic / basal ganglia lesions | 1(5%) |
| Other cystic lesion | 4 (20%) |

**Supplementary Table S2** Focal brain lesions of the infants excluded from the analysis.
